# Detecting Sleep/Wake Rhythm Disruption Related to Cognition in Older Adults With and Without Mild Cognitive Impairment Using the myRhythmWatch Platform: Feasibility and Correlation Study

**DOI:** 10.2196/67294

**Published:** 2025-04-07

**Authors:** Caleb D Jones, Rachel Wasilko, Gehui Zhang, Katie L Stone, Swathi Gujral, Juleen Rodakowski, Stephen F Smagula

**Affiliations:** 1 Primary Care Accelerated Track, School of Medicine, University of Pittsburgh, Pittsburgh, PA, United States; 2 Department of Psychiatry, School of Medicine, University of Pittsburgh, 3811 O'Hara Street, Pittsburgh, PA, 15213, United States; 3 School of Sciences, Southwest Petroleum University, Chengdu, Sichuan, China; 4 California Pacific Medical Center Research Institute, San Francisco, CA, United States; 5 Department of Epidemiology and Biostatistics, University of California, San Francisco, CA, United States; 6 Department of Occupational Therapy, School of Health and Rehabilitation Sciences, University of Pittsburgh, Pittsburgh, PA, United States

**Keywords:** sleep, sleep/wake, circadian, activity pattern, dementia, cognition, mobile sensing, actigraphy, accelerometer

## Abstract

**Background:**

Consumer wearable devices could, in theory, provide sufficient accelerometer data for measuring the 24-hour sleep/wake risk factors for dementia that have been identified in prior research. To our knowledge, no prior study in older adults has demonstrated the feasibility and acceptability of accessing sufficient consumer wearable accelerometer data to compute 24-hour sleep/wake rhythm measures.

**Objective:**

We aimed to establish the feasibility of characterizing 24-hour sleep/wake rhythm measures using accelerometer data gathered from the Apple Watch in older adults with and without mild cognitive impairment (MCI), and to examine correlations of these sleep/wake rhythm measures with neuropsychological test performance.

**Methods:**

Of the 40 adults enrolled (mean [SD] age 67.2 [8.4] years; 72.5% female), 19 had MCI and 21 had no cognitive disorder (NCD). Participants were provided devices, oriented to the study software (myRhythmWatch or myRW), and asked to use the system for a week. The primary feasibility outcome was whether participants collected enough data to assess 24-hour sleep/wake rhythm measures (ie, ≥3 valid continuous days). We extracted standard nonparametric and extended-cosine based sleep/wake rhythm metrics. Neuropsychological tests gauged immediate and delayed memory (Hopkins Verbal Learning Test) as well as processing speed and set-shifting (Oral Trails Parts A and B).

**Results:**

All participants meet the primary feasibility outcome of providing sufficient data (≥3 valid days) for sleep/wake rhythm measures. The mean (SD) recording length was somewhat shorter in the MCI group at 6.6 (1.2) days compared with the NCD group at 7.2 (0.6) days. Later activity onset times were associated with worse delayed memory performance (*β*=−.28). More fragmented rhythms were associated with worse processing speed (*β*=.40).

**Conclusions:**

Using the Apple Watch-based myRW system to gather raw accelerometer data is feasible in older adults with and without MCI. Sleep/wake rhythms variables generated from this system correlated with cognitive function, suggesting future studies can use this approach to evaluate novel, scalable, risk factor characterization and targeted therapy approaches.

## Introduction

Twenty-four-hour sleep/wake characteristics, which are objectively measurable using accelerometer-containing devices, are related to both dementia biomarkers and dementia risk. Prior studies have shown that sleep/wake rhythm disruption, including fragmentation of 24-hour sleep/wake rhythms, temporally precedes the incidence of mild cognitive impairment (MCI) and dementia [1,2]. Even among adults with normal cognition, rhythm fragmentation correlates with greater brain amyloid deposition [3,4]. Over time, 24-hour rhythm fragmentation has been associated with increased risk and faster rates of cognitive decline in people with MCI and mild-to-moderate dementia [1,5] .

The above-mentioned studies linking sleep/wake rhythm disruption with cognitive impairment and neurodegenerative processes in aging raise the potential that accelerometer-based sleep/wake monitoring may have useful clinical applications. For example, wearable accelerometers could potentially be used to identify individuals who have established sleep/wake risk factors for dementia, assign targeted interventions, and track sleep/wake patterns throughout clinical trials. Compared with researcher-focused accelerometer devices, consumer wearables (which also contain triaxial accelerometers) could yield more widely scalable, and clinician and user-friendly, systems for developing and testing potential applications. Our prior pilot study demonstrated that it is possible to collect 24-hour accelerometer data from the Apple Watch and generate standard 24-hour sleep/wake rhythm measures [6]. However, this prior study was limited to a convenience sample of young adults.

Prior to studies evaluating the use of consumer wearable-based sleep/wake monitoring for dementia risk stratification and targeting prevention approaches, we sought to establish the feasibility of using a consumer wearable device to assess 24-hour sleep/wake patterns in older adults (including those with elevated dementia risk by virtue of having a diagnosis of MCI). Our first aim was therefore to evaluate the feasibility of using the Apple Watch and a software platform called myRhythmWatch (myRW) to obtain 24-hour accelerometer data assessing sleep/wake rhythms in older adults with and without MCI. Second, we sought to validate that the sleep/wake rhythm data collected from this system is relevant to cognition. To do so, we evaluated if sleep/wake rhythm variables extracted from this system correlated with cognitive function similar to prior published studies that used researcher-focused accelerometer devices.

## Methods

### Participants and Study Protocol

Participants were identified by referral from either local studies that adjudicated MCI diagnoses or a local research recruitment registry that is led by the University of Pittsburgh Clinical and Translational Science Institute (Pitt+Me). The inclusion criteria were (1) being 50 years of age or older; (2) passing the San Diego Brief Assessment of Capacity to Consent [7] with scores ≥14.5; (3) have a score of >27 on the Telephone Interview for Cognitive Status (TICS) [8]; (4) having a TICS score of either ≤34 (high-risk group) or ≥39 (no cognitive disorder or NCD); and (5) have a prior adjudicated diagnosis of MCI (high-risk group) or reporting no concerns regarding cognitive decline (ie, NCD). The exclusion criteria included (1) self-reporting active behavioral health treatments for insomnia or depression; (2) self-reporting of performing the prescribed exercises; and (3) self-reported use of sleep medications every night or nearly every night. After explaining the study purpose and procedures, we obtained verbal consent to screen interested individuals for eligibility. Of 82 potentially eligible participants who completed the verbal eligibility screening, 35 were ineligible, 6 refused, and 41 were enrolled. One participant withdrew after enrollment, resulting in an analytic sample of 40 individuals (19 high-risk (ie, having a low TICS score and prior MCI diagnosis) and 21 low-risk (ie, having a high TICS score and no prior MCI diagnosis), as shown in Figure S1 in Multimedia Appendix 1 (ie, a flow diagram illustrating how we arrived at our analytic sample).

After completing web-based written informed consent forms, participants completed baseline procedures and assessments via Health Insurance Portability and Accountability Act (HIPPA)-compliant video-conferencing. We sent participants the following study devices: an Apple Watch 8, iPhone SE 2nd generation with active data plans, and nonstock 40-Watt charger. After the participants received the devices, we conducted an additional study visit to instruct them on logging in and using the myRW application. We specifically instructed participants that about 20‐30 minutes of charging a day is sufficient, and asked them to wear the watch whenever it was not charging for a week.

### Ethical Considerations

All study procedures were approved by the University of Pittsburgh Institutional Review Board (identifier: STUDY22080033). All the participants completed web-based written informed consent forms

### Study Application

**Figure 1.**
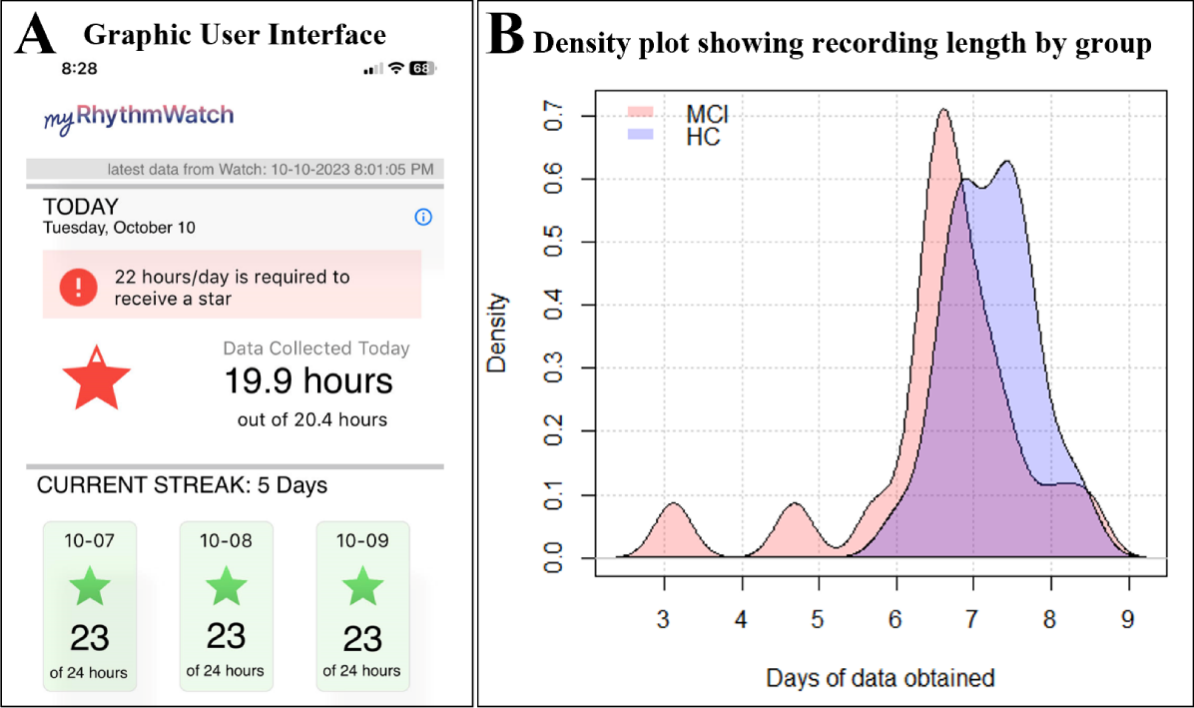
Illustrations of the graphic user interface (left) and the amount of accelerometer data collected in the sample (right). The difference in the mean (SD) recording length between the no cognitive disorder (NCD; 7.2 [0.6]) and mild cognitive impairment (MCI; 6.6 [1.2]) groups is shown visually in the density plot (*P*=.04).

The myRW software system, developed by the corresponding author, extracts triaxial accelerometer data that are recorded at 50 hertz on the Apple Watch. After bandpass filtering, we aggregate across axes and into 30-second counts, and transmit data for storage and further computations on the Amazon Web Services platform. To encourage data collection, we depict the amount of data collected per day graphically by proportionally filling and color coding a star (Figure 1). We explicitly told participants that sufficient data were required to “get a star,” that the goal was to get 7 stars in a row, and we also displayed information on how many days in a row participants obtained stars (to encourage data collection “streaks”). Participants received reminders three times daily to check in to the application. Other features, including personal data graphs and sleep/wake metrics, were disabled for this study to prevent the feedback from potentially altering the users’ behavior/typical sleep/wake patterns.

### Sleep/Wake Measures

Consistent with prior publications using researcher-focused accelerometers [9-11], we required participants to have at least 3 valid continuous days of accelerometer data to consider the recording adequate for processing. Valid days were defined, consistent with National Sleep Research Resource data processing standards [12], as those with no more than 4 hours of nonwear time or any nonwear time during the main sleep period. Invalid days and remaining data missing within days were censored (not imputed). Although we applied this aforementioned 20 hours per day criterion, to maximize data collection, note that the app was programmed to reward participants with a green star only if they collected at least 22 hours of data per day.

Following previously published technical definitions, we calculated extended cosine-based [13] and nonparametric [14-17] sleep/wake measures using the R package ‘RAR’ and custom code. From the extended-cosine models, we extracted measures of 24-hour robustness (pseudo-F statistic, indicating how well the observed data fits the 24-hour curve); activity onset time (up-mesor, the time which the modeled activity level passes the middle modeled rhythm height prior to the peak); and activity offset time (down-mesor or the time which the modeled activity level passes the middle modeled rhythm height prior to the nadir). From the nonparametric method, we calculated the cross-daily stability (inter-daily stability, measuring the consistency of circadian sleep/wake activity rhythms across days); rhythm strength (relative amplitude, measuring the standardized peak-trough difference of 24-hour activity rhythms); and sleep/wake rhythm fragmentation (intradaily variability or IV, measuring the frequency and extent of transitions in activity levels). Although sleep/wake rhythm fragmentation is typically measured using differences in activity levels every hour, it can also be computed on a range of timescales [16,17]. For all nonparametric metrics, we used all-time series data and did not subsample to hourly activity levels, as described previously [14]. To explore the relevance of fragmentation timescale, we computed IV values using numerator timescales ranging from 5 to 60 minutes, but holding the denominator constant as done previously [16].

### Neuropsychological Measures

Cognitive performance was measured using the Hopkins Verbal Learning Test (HVLT; PAR, Lutz, FL) and the Oral Trail Making Test (O-TMT).

The HVLT is a verbal list learning measure with three trials of 12 words from multiple representative semantic categories, and includes delayed recall and delayed recognition memory components [18,19]. For the delayed recall, participants were asked to repeat as many terms as they could recall from the word listed presented to them thrice, 30 minutes earlier. For delayed memory recognition, participants were asked to recognize those same terms appearing on a broader list containing semantically related terms, ie, “horse” must be differentiated from “dog,” which was not part of the original term set. The HVLT assessed encoding, storage, and retrieval of noncontextual verbal information.

Processing speed was measured as the time taken on Part A of the O-TMT, which requires the subject to verbally count upwards from 1 to 25 as quickly as they are able [20]. Set-shifting, an aspect of cognitive function, was measured as the time taken using Part B of the O-TMT, which requires the subject to alternate between listing numbers and letters in sequence (“1, A, 2, B,” etc). Note that longer times on both parts of the O-TMT indicates worse performance [21].

### Statistical Analyses

To compare recording lengths between the MCI and NCD groups, we used an independent sample *t* test and density plots illustrating recording length distributions by groups. To examine relationships of sleep/wake rhythm measures with cognitive function, we first adjusted for key confounders by taking the residuals from linear regression models (one for each cognitive outcome variable) that had the confounders age, sex, education (college degree vs less than college degree), and accelerometer recording length as predictor variables. We used the residualized values as age, sex, education, and recording length-adjusted cognitive outcome variables in a series of linear regressions (one per sleep/wake predictor variable). Since there were many conceptually similar or highly inter-correlated intradaily variability metrics, for related analyses, we only report Benjamini-Hochberg corrected false discovery rates (which are here referred to as *q* values instead of *P* values) [22] to account for the 12 statistical tests relating intradaily variability metrics within each cognitive outcome.

## Results

### Sample Characteristics

The sample included older adults, with a mean (SD) age of 67.2 (8.4) years; the majority of the participants were females with college degrees (Table 1).

**Table 1.** Sample characteristics.

Characteristics	Value
Age, years, mean (SD)	67.2 (8.4)
Female sex, n (%)	29 (72.5)
College degree or greater, n (%)	29 (72.5)
Prior diagnosis of MCI^a^, n (%)	19 (47.5)
Accelerometer recording length, days, mean (SD)	6.9 (0.9)
Immediate memory, mean (SD)	26.7 (5.2)
Delayed memory, mean (SD)	8.7 (2.8)
Processing speed, seconds, mean (SD)	10.3 (2.8)
Set shifting executive function, seconds, mean (SD)	40.1 (31.6)

a MCI: mild cognitive impairment

### Accelerometer Recording Lengths in People With and Without a Diagnosis of MCI

In both the groups, all participants achieved the minimum data requirement for computing 24-hour sleep/wake rhythm assessments. There was, however, a statistically significant difference in the mean (SD) recording lengths of about a half a day when comparing the NCD (7.2 [0.6] days) and MCI groups (6.6 [1.2] days), with the Satterthwaite test assuming unequal group variances (*df* 25.033; *t* value −2.15; *P*=.04). This difference was due to 3 individuals in the MCI group who collected 3‐6 days of data each (Figure 1).

### Associations of Sleep/Wake Characteristics With Cognitive Performance

As shown in Table 2, there were small effect size associations of later activity onset time with lower delayed memory performance (*β*=−.28, 95% CI −0.55 to ‐0.02; *t*=−2.17, *df*=38, *P*=.04) and more stable rhythms with better processing speed and performance (*β*=−0.27, 95% CI: −0.54 to 0.00; *t*=−2.00, *df*=38, *P*=.05). None of the other sleep/wake measures listed in Table 2 were associated with the cognitive outcomes. Regarding sleep/wake rhythm fragmentation, greater fragmentation levels in the 40‐60-minute timeframe was significantly associated with worse processing speed and performance (Table 3; β point estimate range: 0.36, 0.40; *q*=.022). When repeating analyses in the subgroup with at least 6 days of data, results were not substantively altered (n=37; see Tables S1 and S2 in Multimedia Appendix 1).

**Figure 2.**
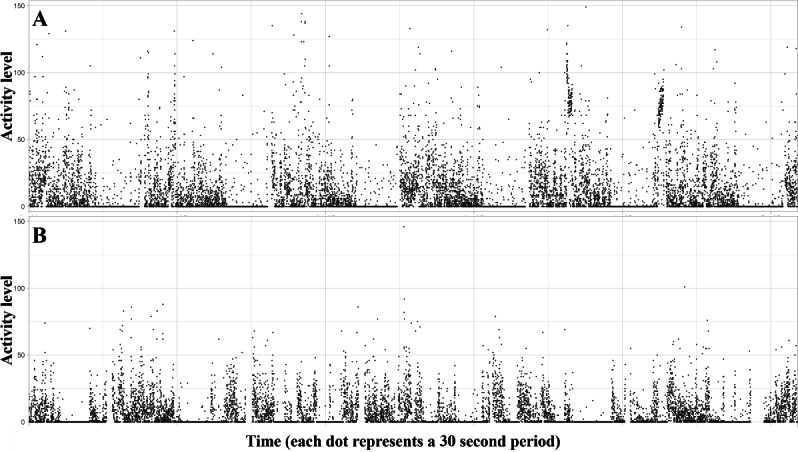
Accelerometer data from two participants. Top: The participant was in the low risk (no cognitive disease) group and had relatively lower sleep/wake rhythm fragmentation. Bottom: The participant is from the high-risk (mild cognitive impairment) group and had relatively higher sleep/wake rhythm fragmentation.

To illustrate the accelerometer data collected in this study and visualize 24-hour sleep/wake fragmentation, Figure 2 shows data from example participants with lower and higher degrees of 24-hour sleep/wake rhythm fragmentation.

**Table 2.** Associations between 24-hour sleep/wake rhythm variables and cognitive performance.

Variable	Immediate memory^a^		Delayed memory^a^		Psychomotor speed/attention^b^		Set shifting executive function^b^	
	*β* (95% CI)	*P* value	*β* (95% CI)	*P* value	*β* (95% CI)	*P* value	*β* (95% CI)	*P* value
24 h robustness	.06 (−.22 to .34)	.67	.17 (−.11 to 0.44)	.22	−.09 (−.38 to .20)	.53	−.18 (−.48 to .11)	.22
Cross-daily stability	.11 (−.17 to .39)	.44	.13 (−.14 to .41)	.34	−.27 (−.54 to .00)	.05	−.11 (−.42 to .19)	.45
Rhythm strength	.08 (−.21 to .36)	.59	.04 (−.23 to .32)	.75	−.04 (−.33 to .24)	.76	−.12 (−.42 to .18)	.43
Activity onset time	−.13 (−.41 to .15)	.36	−.28 (−.55 to ‐.02)	.04	.04 (−.24 to .33)	.76	−.11 (−.41 to .19)	.46
Activity offset time	−.03 (−.32 to .25)	.81	−.07 (−.35 to .21)	.60	−.12 (−.41 to .16)	.40	.17 (−.13 to .47)	.26

a Higher scores on the memory tests indicates better performance (as the outcome is number of items recalled)

b For psychomotor speed/attention and set-shifting, higher scores indicate worse performance (as the outcome is the duration of time to complete Oral Trails A and B, respectively)

**Table 3.** Associations between 24-hour sleep/wake rhythm fragmentation on various timescales with cognitive performance.

Time scale	Immediate memory^a^		Delayed memory^a^		Psychomotor speed/attention^b^		Set shifting executive function^b^	
	*β* (95% CI)	*q* value	*β* (95% CI)	*q* value	*β* (95% CI)	*q* value	*β* (95% CI)	*q* value
5 minutes	-.10 (−.38 to .18)	0.938	−.01 (−.29 to .27)	0.938	.14 (−.14 to .43)	0.314	.28 (−.01 to .58)	0.092
10 minutes	−.10 (−.38 to .18)	0.938	−.02 (−.3 to .26)	0.938	.14 (−.14 to .43)	0.314	.26 (−.03 to .55)	0.102
15 minutes	−.10 (−.39 to .18)	0.841	−.05 (−.33 to .23)	0.841	.16 (−.12 to .45)	0.298	.30 (.02 to .59)	0.092
20 minutes	−.12 (−.41 to .16)	0.628	−.11 (−.38 to .17)	0.628	.21 (−.07 to .49)	0.203	.34 (.06 to .62)	0.092
25 minutes	−.11 (−.40 to .17)	0.628	-.10 (-.38 to .18)	0.628	.20 (−.08 to .48)	0.203	.34 (.06 to .63)	0.092
30 minutes	−.11 (−.39 to .17)	0.628	−.11 (−.39 to .16)	0.628	.23 (−.05 to .51)	0.177	.32 (.03 to .61)	0.092
35 minutes	−.09 (−.37 to .19)	0.628	−.13 (−.41 to .14)	0.628	.28 (.01 to .55)	0.085	.28 (−.01 to .57)	0.093
40 minutes	−.08 (−.36 to .20)	0.628	−.15 (−.42 to .13)	0.628	.37 (.10 to .63)	0.022	.32 (.03 to .60)	0.092
45 minutes	-.09 (-.38 to .19)	0.628	−.16 (−.44 to .11)	0.628	.37 (.11 to .63)	0.022	.29 (.00 to .58)	0.092
50 minutes	−.11 (−.39 to .17)	0.628	−.18 (−.45 to .09)	0.628	.40 (.14 to .65)	0.022	.25 (−.05 to .54)	0.118
55 minutes	−.08 (−.36 to .21)	0.628	−.18 (−.45 to .1)	0.628	.36 (.09 to .62)	0.022	.17 (−.13 to .47)	0.254
60 minutes	−.12 (−.40 to .16)	0.628	−.23 (−.50 to .04)	0.628	.36 (.09 to .62)	0.022	.18 (−.12 to .48)	0.250

a Higher scores on the memory tests indicates better performance (as the outcome is number of items recalled).

b For psychomotor speed/attention and set-shifting, higher scores indicate worse performance (as the outcome is duration of time to complete Oral Trails A and B, respectively).

## Discussion

These results demonstrate that it is feasible to collect raw accelerometer data and characterize 24-hour sleep/wake rhythms in older adults with and without MCI using the Apple Watch and myRW system. Each individual in our sample met the minimum data requirement to derive sleep/wake rhythms measures. Notably, however, several individuals in the MCI group generated shorter recordings. Thus, while supporting the feasibility of using the Apple Watch and myRW system to assess sleep/wake rhythms in people with MCI who are at risk for dementia, our findings also suggest that some individuals with MCI may require additional support (eg, additional human support or programmed reminders) and data imputation [23] to monitor sleep/wake rhythms over longer periods with this system.

With regard to our second aim, we found that sleep/wake rhythm variables extracted from the Apple Watch accelerometer data were correlated with cognitive function. We found small-to-medium effect size correlations between sleep/wake measures and cognitive performance. Specifically, we found that later activity onset times were related to worse delayed memory performances. In addition, less stable and more fragmented rhythms were correlated with worse processing speed. Detecting these signs of disrupted sleep/wake rhythms early on could help target prevention strategies, given prior research demonstrating that sleep/wake rhythm fragmentation [1], worse memory [24,25], and slower processing speeds [26] are all associated with the risk of developing dementia.

The study had several limitations. This was a cross-sectional observational study limited to accelerometer and neuropsychological measures, so there is no way to determine causality or the mechanisms underlying associations between the variables examined. The sample size was relatively small; therefore, there is a risk of false negative associations and effect size estimates (relating sleep/wake measures with cognition) that should be deemed less reliable than those from large epidemiologic studies. We failed to detect some associations that were expected based on prior literature examining dementia risk (eg, previous findings linking low rhythm strength with dementia risk [27]). Larger studies examining the relationships between these sleep/wake factors and specific domains of cognitive function, earlier in the disease processes, will be needed to verify our findings. We made efforts to minimize data loss as described above, but did not use data imputation, which could be applied in future studies. Additionally, given the small sample, future studies will be required to confirm results of using this system in samples that are more broadly representative, eg, samples including more ethnically diverse population subgroups. Finally, we note that determining the mechanism underlying these relationships between sleep/wake rhythm disruption, cognition, and dementia risk is outside the scope of this work. Recent literature has notably suggested that sleep/wake rhythm fragmentation relates to neurodegeneration of the locus coeruleus [28], which could hasten dementia pathology and cognitive decline [29,30].

In summary, we have demonstrated that it is feasible to use the Apple Watch and myRW system to gather accelerometer data and characterize sleep/wake risk factors for dementia in older adults including adults with MCI. A strength of this study is the use of the Apple Watch, which is already voluntarily being used by millions of people, as it may provide increased scalability for applications of sleep/wake risk factor monitoring into the general public and general practice settings. One implication of this research is that it is feasible for future research to be conducted for evaluating if monitoring sleep/wake disruption using consumer wearable-based systems improves upon existing dementia risk factor detection and management approaches. Future studies will also be needed to examine if tailoring interventions using information on sleep/wake patterns derived from this system improves outcomes among older adults who are at risk for dementia.

## Supplementary material

10.2196/67294 Multimedia Appendix 1 Supplementary Tables 1 and 2 and Figure 1.

## Abbreviations

HVLT: Hopkins Verbal Learning Test
MCI: mild cognitive impairment
NCD: no cognitive disorder
O-TMT: Oral Trail Making Test
