# Supplementary material for: Detecting Sleep/Wake Rhythm Disruption Related to Cognition in Older Adults With and Without Mild Cognitive Impairment Using the myRhythmWatch Platform: Feasibility and Correlation Study
Source: JMIR Aging. 2025 Apr 7;8:e67294. doi: 10.2196/67294 (PMC11996143; doi:10.2196/67294)
Supplement: Multimedia Appendix 1 [file aging-v8-e67294-s001.docx]

| **Supplemental Table 1.** Associations between 24-hour sleep/wake rhythm variables and cognitive performance restricting to participants with at least 6 days of data (n=37) | | | | | | | | |
| --- | --- | --- | --- | --- | --- | --- | --- | --- |
|  | Immediate memory | | Delayed memory | | Psychomotor speed/attention | | Set shifting executive function | |
|  | β (95% CI) | p | β (95% CI) | p | β (95% CI) | p | β (95% CI) | p |
| 24-hour robustness | 0.07 (-0.26, 0.39) | 0.67 | 0.16 (-0.15, 0.48) | 0.31 | -0.07 (-0.39, 0.25) | 0.67 | -0.18 (-0.52, 0.16) | 0.29 |
| Cross-daily stability | 0.11 (-0.19, 0.40) | 0.46 | 0.13 (-0.16, 0.42) | 0.36 | **-0.27 (-0.54, 0.01)** | **0.06** | -0.11 (-0.42, 0.20) | 0.47 |
| Rhythm strength | 0.05 (-0.25, 0.35) | 0.73 | 0.03 (-0.26, 0.33) | 0.83 | -0.09 (-0.39, 0.20) | 0.52 | -0.09 (-0.41, 0.22) | 0.57 |
| Activity onset time | -0.23 (-0.59, 0.11) | 0.19 | **-0.42 (-0.75, -0.09)** | **0.01** | 0.02 (-0.33, 0.38) | 0.91 | -0.18 (-0.56, 0.20) | 0.36 |
| Activity offset time | 0.02 (-0.33, 0.37) | 0.91 | -0.10 (-0.45, 0.23) | 0.55 | -0.05 (-0.39, 0.29) | 0.75 | 0.20 (-0.17, 0.56) | 0.28 |
| Note that higher scores on the memory tests indicates better performance (as the outcome is number of items recalled). However, for psychomotor speed/attention and set-shifting, higher scores indicate worse performance (as the outcome is duration of time to complete Oral Trails A and B, respectively) | | | | | | | | |

| **Supplemental Table 2.** Associations between 24-hour sleep/wake rhythm fragmentation on various timescales with cognitive performance restricting to participants with at least 6 days of data (n=37) | | | | | | | | |
| --- | --- | --- | --- | --- | --- | --- | --- | --- |
|  | Immediate memory | | Delayed memory | | Psychomotor speed/attention | | Set shifting executive function | |
|  | β (95% CI) | q | β (95% CI) | q | β (95% CI) | q | β (95% CI) | q |
| 5 minutes | -0.14 (-0.45, 0.16) | 0.588 | -0.01 (-0.32, 0.29) | 0.938 | 0.14 (-0.14, 0.43) | 0.314 | 0.28 (-0.01, 0.58) | 0.093 |
| 10 minutes | -0.16 (-0.47, 0.15) | 0.588 | -0.02 (-0.34, 0.29) | 0.938 | 0.14 (-0.14, 0.43) | 0.314 | 0.26 (-0.03, 0.55) | 0.103 |
| 15 minutes | -0.15 (-0.46, 0.16) | 0.588 | -0.05 (-0.36, 0.26) | 0.841 | 0.16 (-0.12, 0.45) | 0.298 | 0.30 (0.02, 0.59) | 0.093 |
| 20 minutes | -0.19 (-0.5, 0.13) | 0.588 | -0.11 (-0.43, 0.2) | 0.628 | 0.21 (-0.07, 0.49) | 0.203 | 0.34 (0.06, 0.62) | 0.093 |
| 25 minutes | -0.14 (-0.45, 0.16) | 0.588 | -0.09 (-0.40, 0.21) | 0.628 | 0.20 (-0.08, 0.48) | 0.203 | 0.34 (0.06, 0.63) | 0.093 |
| 30 minutes | -0.15 (-0.46, 0.16) | 0.588 | -0.11 (-0.42, 0.2) | 0.628 | 0.23 (-0.05, 0.51) | 0.177 | 0.32 (0.03, 0.61) | 0.093 |
| 35 minutes | -0.13 (-0.44, 0.18) | 0.588 | -0.13 (-0.44, 0.17) | 0.628 | 0.28 (0.01, 0.55) | 0.086 | 0.28 (-0.01, 0.57) | 0.093 |
| 40 minutes | -0.11 (-0.43, 0.21) | 0.588 | -0.15 (-0.46, 0.16) | 0.628 | **0.37 (0.1, 0.63)** | **0.022** | 0.32 (0.03, 0.60) | 0.093 |
| 45 minutes | -0.11 (-0.42, 0.19) | 0.588 | -0.16 (-0.46, 0.14) | 0.628 | **0.37 (0.11, 0.63)** | **0.022** | 0.29 (0.00, 0.58) | 0.093 |
| 50 minutes | -0.13 (-0.45, 0.18) | 0.588 | -0.18 (-0.49, 0.12) | 0.628 | **0.40 (0.14, 0.65)** | **0.022** | 0.25 (-0.05, 0.54) | 0.118 |
| 55 minutes | -0.1 (-0.4, 0.21) | 0.588 | -0.18 (-0.47, 0.12) | 0.628 | **0.36 (0.09, 0.62)** | **0.022** | 0.17 (-0.13, 0.47) | 0.254 |
| 60 minutes | -0.13 (-0.43, 0.17) | 0.588 | -0.22 (-0.51, 0.07) | 0.628 | **0.36 (0.09, 0.62)** | **0.022** | 0.18 (-0.12, 0.48) | 0.251 |
| Note that higher scores on the memory tests indicates better performance (as the outcome is number of items recalled). However, for psychomotor speed/attention and set-shifting, higher scores indicate worse performance (as the outcome is duration of time to complete Oral Trails A and B, respectively) | | | | | | | | |

**Supplemental Figure 1.** Flow diagram illustrating how we arrived at our analytic sample.

3 obtained <6 days of data

37 included in sensitivity analysis:

- 16 high-risk
- 21 low-risk

35 ineligible

6 refused

1 withdrew after enrollment

40 completed study procedures:

- 19 high-risk
- 21 low-risk

41 enrolled

82 individuals screened
